# Restoring colistin sensitivity in colistin-resistant *E. coli*: Combinatorial use of MarR inhibitor with efflux pump inhibitor

**DOI:** 10.1038/s41598-019-56325-x

**Published:** 2019-12-25

**Authors:** Niranjana Sri Sundaramoorthy, Pavithira Suresh, Subramaniapillai Selva Ganesan, ArunKumar GaneshPrasad, Saisubramanian Nagarajan

**Affiliations:** 1 Center for Research on Infectious Diseases, School of Chemical and Biotechnology, SASTRA Deemed to be University, Thanjavur, Tamil Nadu India; 2 Department of Chemistry, School of Chemical and Biotechnology, SASTRA Deemed to be University, Thanjavur, Tamil Nadu India

**Keywords:** Microbiology, Antimicrobial resistance

## Abstract

Antibiotics like colistin are the last resort to deal with infections by carbapenem-resistant *Enterobacteriaceae* (CREB). Resistance to colistin severely restricts therapeutic options. To tackle this dire situation, urgent measures to restore colistin sensitivity are needed. In this study, whole-genome sequencing of colistin-resistant *E. coli* strain was performed and the genome analysis revealed that the strain belonged to the sequence type ST405. Multiple mutations were observed in genes implicated in colistin resistance, especially those related to the L-Ara-4-N pathway but *mgrB* was unmutated and *mcr1-9* genes were missing. MarR inhibitor salicylate was used to re-sensitize this strain to colistin, which increased the negative charge on the cell surface especially in colistin resistant *E. coli* (U3790 strain) and thereby facilitated a decrease in colistin MIC by 8 fold. It is indeed well known that MarR inhibition by salicylate triggers the expression of AcrAB efflux pumps through MarA. So, in order to fully restore colistin sensitivity, a potent efflux pump inhibitor (BC1), identified earlier by this group was employed. The combination of colistin with both salicylate and BC1 caused a remarkable 6 log reduction in cell counts of U3790 in time-kill assay. Infection of muscle tissue of zebrafish with U3790 followed by various treatments showed that the combination of colistin + salicylate + BC1 was highly effective in reducing bioburden in infected muscle tissue by 4 log fold. Thus, our study shows that a combination of MarR inhibitor to enhance colistin binding and efflux pump inhibitor to reduce colistin extrusion was highly effective in restoring colistin sensitivity in colistin-resistant clinical isolate of *E. coli in vitro* and *in vivo*.

## Introduction

Antimicrobial resistance (AMR) poses a grave threat to public health and is attributed as the third major cause of mortality worldwide. Rise in infections caused by multidrug-resistant (MDR) pathogens prompted WHO to declare a list of 12 priority AMR pathogens in 2017, of which, carbapenem-resistant *Enterobacteriaceae* (*Escherichia coli, Klebsiella spp, Serratia spp, Proteus spp*) fall under critical priority group^1^. Due to rising AMR menace, new antimicrobial agents or resistance modulatory agents to curtail AMR are urgently required. Recently, WHO has released its first report on AMR surveillance, which reveals an alarming trend of widespread dissemination of resistant traits among microbes globally, wherein resistance to commonly used antibiotics were found to range from 0–82%^2^.

Carbapenem-resistant *Enterobacteriaceae* (CREB) is becoming resistant to almost all antibiotics and its clinical outcomes are also poor^3,4^. Among *Enterobacteriaceae, Escherichia coli* tops the list in causing a wide range of clinical infections due to their high prevalence, multidrug resistance and most importantly, rapid acquisition/ transfer of resistance traits by horizontal gene transfer^5,6^. Colistin is a drug of last resort for CREBs^7^. Colistin resistance implies a pan drug resistant state, with virtually no therapeutic options. The situation gets exacerbated by the fact that colistin resistance mediated by *mcr1-9* genes get rapidly disseminated across diverse bacteria by horizontal gene transfer^8–12^. Therefore, alternate approaches to target colistin resistance in these extremely drug resistant (XDR) *Enterobacteriaceae* isolates are urgently required.

Multiple antibiotic resistance (*mar*) locus was earlier identified as a determinant for resistance to major antibiotic classes like quinolones, β-lactams, and tetracyclines^13,14^. A very recent study has elegantly shown that a large subset of antibiotic-resistant clinical strains of *E. coli* exhibit mutation in *marR* loci resulting in de-repression of *marA*. Apart from activating AcrAB efflux transporters, *marA* was also shown to activate *waaY* gene, which alters the charge on LPS, thus, causing collateral sensitivity to cationic peptides in drug-resistant clinical strains^15^. Because colistin also interacts with bacterial cell surface depending on its electronic charge, we explored whether MarR inhibitor (salicylate) would partially restore colistin sensitivity in colistin resistant clinical isolate of *E. coli*. Since MarR inhibition could be counterproductive by leading to enhanced antibiotic resistance predominantly through AcrAB-TolC pump, in the present work, we have also evaluated combination of MarR inhibitor along with a non – toxic efflux pump inhibitor (benzochromenes) identified earlier by our group (against NorA pump of *S. aureus*)^16^, to fully restore colistin sensitivity in colistin-resistant *E. coli in vitro* and *in vivo*.

## Results

### Antimicrobial activity

The studies were performed with six *E. coli* clinical isolates obtained from a tertiary care hospital, Chennai, India and enteropathogenic and enterotoxigenic *E. coli* strains obtained as a kind.gift from Prof. T. Ramamurthy, THSTI, India. *E. coli* MG1655 was used as a reference strain. Among the strains employed, an *E. coli* clinical isolate designated as U3790 (isolated from urine of infected child) was found to display resistance to a wide range of antimicrobials including colistin and hence became our strain of interest. In a previous study, we reported antimicrobial profiling of U3790 against diverse antimicrobial agents^17^. Hence, the antimicrobial effect of colistin, salicylate, and BC1 (benzochromene derivative) was evaluated against all strains employed in the present study (Supplementary Table 1). The results showed that the clinical isolate of *E. coli* (U3790) was highly resistant to colistin with an MIC of 32 µg/ml. The other clinical isolate U1007 was slightly resistant to colistin (MIC 4 µg/ml). Remaining isolates were sensitive to colistin with MIC ≤ 2 µg/ml. Tests on other compounds displayed a higher MIC of 10 mM (salicylate), >256 µg/ml (BC1) and 128 µg/ml (CCCP) against all strains.

### Whole-genome sequence analysis – resistant factors determination

As the clinical *E. coli* strain U3790 was extremely drug-resistant, to understand its resistome, we performed whole-genome sequencing using Illumina platform (HiSeq 2500) at NCBS, Bengaluru, India, and the raw sequence reads were submitted to SRA database of NCBI (Acc No PRJNA541219). Reference guided assembly using *E. coli* MG1655 sequence was performed using BWA program. The assembled genome was annotated using the RAST server. Bacterial analysis pipeline tool of center for genomic epidemiology showed that strain belonged ST405 sequence type and the closest match in terms of sequence homology (65% template coverage) was observed with *E. coli* UMN026 strain. Earlier reports have shown that ST405 strains are extra-intestinal pathogenic *E. coli* known for its multiple antibiotic resistance genes and unique NDM-4 carbapenemase gene flanked by IS26 elements^18^. To identify antimicrobial-resistant genes, U3790 genome sequence was provided as input to comprehensive antibiotic resistance database (CARD). Resistance gene identifier (RGI) tool of CARD revealed that at least 52 genes implicated in antibiotic resistance were present in U3790 strain (Supplementary Table 2), making it extremely drug-resistant strain. Most importantly the RGI program revealed that, out of 52 resistance genes, 39 of them were related to antibiotic efflux (Supplementary Fig. 1a and Supplementary Table 2) underscoring the importance of drug efflux in antibiotic resistance. To understand the role of efflux pumps in the drug resistance of U3790, RT PCR for efflux transporter genes *acrA, acrB*, and *tolC* was performed using normalized concentration of cDNA from U3790, colistin sensitive *E. coli* (U3176) and the reference *E. coli* strain MG1655. The results showed that among the RND transporter components, the expression of periplasmic subunit (*acrA*) was 2 fold higher in U3790 relative to both MG1655 and U3176 strain (Supplementary Fig. 1b). But, the expression of the inner membrane component (*acrB*) and the outer membrane component (*tolC*) remained similar in all three strains.

Genome analysis using RAST server revealed that the following genes (*ugd, arnA_DH, arnA_FT, pmrG*) responsible for alteration of cell surface charge of LPS by L-Ara4-N pathway were present in U3790 strain, which might be responsible for enhanced colistin resistance observed in this strain. Although mutations in many other genes involved in colistin resistance like *pmrA, pmrB, pmrC, pmrH, pmrG, phoP, marR, arnT, arnC*, and *parC* were observed in U3790 strain (Supplementary Table 3), some of these mutations were not exclusively limited to colistin-resistant strains and, in fact, few of these mutations were hitherto not reported. The role of these unreported mutations in colistin resistance will be explored in a future study. In addition, attempts to find *mcr1* to *mcr 9* genes in the genome/plasmid sequence of U3790 were unsuccessful. Since *mgrB* is unmutated and observed mutations in colistin-resistant genes were not exclusively restricted to colistin-resistant strains, L-Ara4-N modification pathway might be responsible for colistin resistance in this strain.

As regards the master regulators, *acrR* regulates the expression of *acrAB* operon, *soxS* and *soxR* govern the superoxide response regulon. MarA, SoxR and SoxS regulons also principally regulate the expression of AcrAB-TolC and *micF* (downregulates OmpF)^19,20^. MarA, being a master regulator, is known to affect around 60 chromosomal genes in *E. coli* responsible for different functions (Supplementary Table 4)^21^ mutations in these genes lead to overexpression of *acrAB*, alters the antibiotic target and reduces membrane permeability to antibiotics. All of these ultimately cause the strain to gain resistance to multiple classes of antibiotics (Supplementary Table 5).

### Zeta potential studies

Lazar *et al*.^15^ showed that clinical strains of *E. coli* typically harbor a mutation in *marR* resulting in the upregulation of *waaY*, which increases the cell surface negative charge on LPS by phosphorylation. Taking cue from this study, we explored if chemical inhibition of MarR using salicylate would alter cell surface charge in *E. coli*. Towards this end, we measured cell surface charge of colistin resistant and colistin sensitive *E. coli* in the presence and absence of salicylate using a zeta potential analyzer as reported earlier^22^. Zeta potential measurements showed that even among untreated controls, resistant strains, in general, displayed an overall reduced cell surface charge which is half of the cell surface charge observed in sensitive strains and might account for varying susceptibility to colistin. Interestingly, salicylate treatment caused an increase in cell surface negative charge only in colistin resistant strain of *E. coli*, but not in colistin sensitive strain (U3176) or the reference strain (Table 1). Thus, MarR inhibition by salicylate is likely to alter colistin susceptibility patterns predominantly in colistin resistant strains.

**Table 1:** Colistin resistant strains respond well to the MarR inhibitor by altering cell surface charge.

Strains	Groups	Zeta potential (mV)
U3790	UT	−29.8
	Salicylate Treated (5 mM)	−36.6
U3176	UT	−36.5
	Salicylate Treated (5 mM)	−39.5
MG1655	UT	−48.2
	Salicylate Treated (5 mM)	−46.4

Resistant and reference strains of *E. coli* (10^9^ cells) were treated with 5 mM of salicylate for 20 min at 37 °C, harvested and 10^8^ cells were measured to obtain the zeta potential.

### MIC reversal and synergy

**Figure 1:**
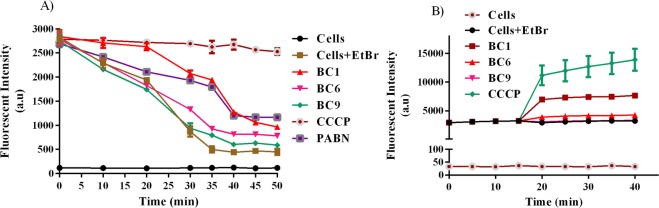
BC1 inhibits efflux and shows increased accumulation of EtBr in colistin resistant clinical isolate of *E. coli*. (**A)** Mid log cells of colistin resistant *E. coli* isolate U3790 were de-energized with CCCP (100 µM) for 20 min. The cells were then harvested, washed and incubated with different benzochrome derivatives (16 µg/ml) along with EtBr for an hour. The cells were washed again and energized in buffer containing glucose. Efflux of EtBr was recorded for 25 min at 5 min intervals. (**B)** Mid log cells were harvested in buffer and allowed to incubate with EtBr for 30 min and BC derivatives were added at 16 µg/ml. Fluorescence was measured for a period of 25 min at 5 min intervals. The experiments were performed in triplicates.

MIC of these strains was determined in the presence and absence of salicylate in order to evaluate whether altered cell surface charge in colistin resistant *E. coli*, mediated by salicylate (MarR inhibitor), translates to reduced colistin MIC. In concurrence with zeta potential measurements, the fold change in colistin MIC (expressed as modulation factor) due to salicylate treatment, for the sensitive strains was found to be insignificant and it remained between 1–2 fold, whereas, the colistin resistant strain displayed a significant drop in MIC by 8 fold (Table 2). Salicylate induced reduction in colistin MIC observed in resistant strains was not quite drastic as expected. The reason for this is that MarR inhibition triggers *marA* expression^23^, which upregulates antibiotic resistance phenotype predominantly through efflux pumps like AcrAB-TolC. Hence, we hypothesized that simultaneous inhibition of both MarR (with salicylate) and efflux (using appropriate efflux pump inhibitor) would result in resensitizing colistin resistant *E. coli* to colistin. To test this hypothesis at first, we evaluated the ability of benzochromenes (reported earlier by us against NorA pump of *S. aureus*^16^), to inhibit EtBr efflux in colistin resistant *E. coli* (U3790). Our observations (Fig. 1A) showed that among the BC derivatives tested, BC1 effected delayed extrusion of EtBr and the trend resembled that of CCCP for first 15 minutes but at later time points, residual EtBr level in BC1 treated cells reduced further relative to CCCP treatment, this could be attributed to the fact that EtBr is a substrate of multiple efflux pumps and CCCP as a protonophore inhibits multiple efflux transporters simultaneously. We also performed time-dependent accumulation of EtBr which displayed a trend similar to CCCP treatment, wherein, addition of BC1 but not BC6/BC9 caused enhanced EtBr accumulation (Fig. 1B), implying that among benzochromene derivatives, only BC1 inhibits efflux transport in *E. coli*. To qualitatively prove efflux inhibition, we performed a cartwheel assay as reported earlier^24^ and the results revealed that among the compounds tested only BC1 and CCCP inhibited EtBr efflux and facilitated intracellular EtBr accumulation (Supplementary Fig. 2)

**Table 2:** Combination of colistin, salicylate and BC1 reverses colistin MIC in colistin resistant bacteria (MF-Modulation factor).

Strains	MIC of colistin (µg/ml)	Colistin + Sal		Colistin + BC1		Colistin + CCCP		Colistin + Sal + BC1		Colistin + Sal + CCCP	
		MIC (µg/ml)	MF	MIC (µg/ml)	MF	MIC (µg/ml)	MF	MIC (µg/ml)	MF	MIC (µg/ml)	MF
U3790	32	4	**8**	2	**16**	4	**8**	0.25	**128**	0.25	**128**
U1007	4	2	**2**	0.5	**8**	0.25	**16**	0.5	**8**	0.25	**16**
U2354	2	1	**2**	0.5	**4**	0.25	**8**	0.25	**8**	0.25	**8**
U1024	2	1	**2**	0.5	**4**	0.125	**16**	0.25	**8**	0.25	**8**
IDH09519	2	1	**2**	0.5	**4**	0.125	**16**	0.25	**8**	0.25	**8**
IDH07933	2	1	**2**	0.5	**4**	0.125	**16**	0.25	**8**	0.25	**8**
MG1655	2	2	**1**	0.062	**32**	2	**1**	0.062	**32**	2	**1**
U3176	1	1	**1**	0.25	**4**	0.5	**2**	0.25	**4**	0.5	**2**

Next, we checked the ability of BC1 alone as an efflux inhibitor to reduce colistin MIC in all the strains employed in this study. When BC1 (16 µg/ml) was combined with colistin, a drastic 16 fold reduction in colistin MIC was observed for U3790. Even for the sensitive strains, BC1 caused a 4–32 fold drop in MIC (Table 2). Positive control CCCP (standard protonophore which inhibits multiple efflux pumps by abolishing proton motive force) reduced colistin MIC in U3790 by 8 fold and the reduction in MIC for the rest of the strains ranged between 2–16 fold.

We were interested to evaluate whether BC1 altered either outer or inner membrane permeability to afford increased intracellular access to colistin. Towards this end, we performed NPN uptake assay to determine outer membrane permeability. Data from NPN uptake assay (Supplementary Fig. 3a) revealed that BC1 does not possess outer membrane permeabilizing effect even at 2X MEC (32 µg/ml), whereas, the NPN uptake factor of colistin and CTAB was two and four-fold higher relative to untreated and BC1 treated groups respectively. To discern the effect of BC1 on inner membrane permeabilization, we also performed a qualitative PI uptake assay by fluorescence microscopy. The results showed that BC1 does not alter inner membrane permeability, as the proportion of PI accumulated cells upon BC1 treatment was negligible relative to CTAB treatment (Supplementary Fig. 3b).

**Figure 2:**
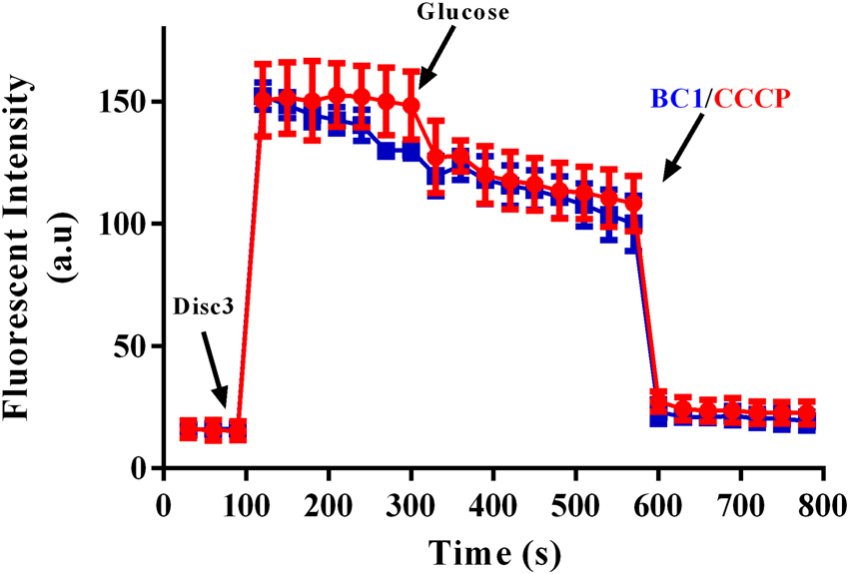
BC1 perturbs membrane potential similar to CCCP. Disc3 was added to mid log cells of U3790 and fluorescence (Ex 622 nm and Em 670 nm) was recorded until plateau. 0.5% glucose was added and fluorescence was again monitored. BC1 and CCCP was added, which led to sudden decline in the fluorescence intensity. The experiment was performed in triplicates and the error bar represents standard error of the mean.

In order to understand the mechanism of efflux inhibition mediated by BC1 (either by direct interaction with the pump or by disrupting proton motive force), membrane potential studies using DiSc3 was performed prior to and post energization with glucose. The results (Fig. 2 and Supplementary Fig. 4) revealed that BC1 displayed a trend similar to CCCP wherein after stabilization of Disc3 fluorescence, glucose addition caused a slight decline in fluorescence, but upon addition of either CCCP/BC1, a marked decline in DiSc3 fluorescence was observed implying that, PMF reestablished by glucose was disrupted by CCCP/BC1, resulting in dye partitioning back to lipid bilayer. Thus, BC1 was able to reduce colistin MIC in most of the strains by indirectly abolishing energy for the pumps that are required to extrude colistin (Table 2).

We further evaluated the ability of BC1 in combination with salicylate to restore colistin sensitivity in different strains of *E. coli*. Interestingly, for most of the strains of *E. coli*, combination of colistin with both salicylate (MarR inhibitor) and BC1 (efflux pump inhibitor) did not induce any further reduction in colistin MIC, relative to decline observed with BC1 + colistin combination (Table 2) implying that inhibition of colistin efflux by BC1 is powerful enough in reducing colistin MIC rather than enhancing colistin binding to cell surface by salicylate treatment. Among the strains that displayed a drop in MIC due to triad treatment (colistin + salicylate + BC1), the most prominent was U3790, which displayed a modulation factor of 128 fold. Colistin + Sal + CCCP was equally effective like Col + Sal + BC1 except in MG1655 strain, where BC1 combination was effective and CCCP combination was ineffective. Overall, the trend shows that Col + Sal + BC1 combination was relatively better than Col + Sal + CCCP in restoring colistin sensitivity in all isolates tested, implying the effectiveness of this combination therapy.

Since colistin MIC was drastically reduced due to combination therapy, we tested whether salicylate and BC1 exhibited synergistic interactions with colistin against both the reference strains and clinical isolates of *E. coli* by checkerboard assay. The results showed that for almost all strains except MG1655, BC1 displayed synergy with colistin and FIC indices were < 0.5 (Table 3). Similarly, CCCP exhibited synergy with colistin against most strains, except for two of the strains U3176 and MG1655.

**Table 3:** BC1 but not salicylate synergizes with colistin against clinical isolates of *E. coli*.

Strains	Col + Sal		Col + BC1		Col + CCCP	
	FIC Index	Effect	FIC Index	Effect	FIC Index	Effect
U3790	0.25	**Synergy**	0.25	**Synergy**	0.20	**Synergy**
U1007	0.62	Additive	0.07	**Synergy**	0.14	**Synergy**
U2354	0.62	Additive	0.25	**Synergy**	0.20	**Synergy**
U1024	0.62	Additive	0.14	**Synergy**	0.14	**Synergy**
IDH09519	0.62	Additive	0.13	**Synergy**	0.14	**Synergy**
IDH07933	0.62	Additive	0.03	**Synergy**	0.14	**Synergy**
MG1655	2	Additive	1.03	Additive	1.07	Additive
U3176	1.07	Additive	0.32	**Synergy**	0.57	Additive

### Colistin accumulation studies

**Figure 3:**
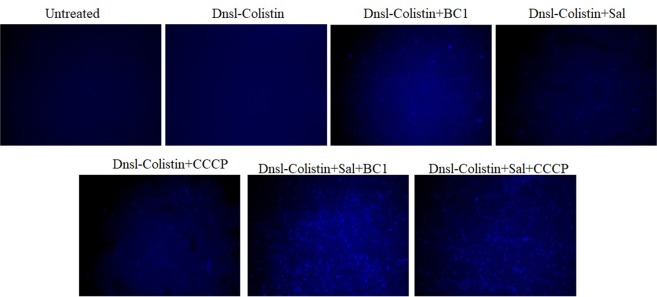
Dansylated colistin accumulation in U3790 is enhanced by salicylate + BC1. Mid log cells were incubated with colistin-dansyl chloride and various treatments were given for 3 h, the cells were washed with sterile PBS and imaged using a fluorescent microscope. CCCP was used as a positive control.

To validate the effect of BC1 alone and salicylate + BC1 on colistin efflux, we dansylated colistin (Dnsl-colistin) and checked its intracellular accumulation, which can be qualitatively analyzed by imaging using a fluorescent microscope. The cells were exposed to Dnsl-colistin in the presence and absence of salicylate/BC1/Sal + BC1. The presence of salicylate along with BC1 resulted in enhanced accumulation of dansylated colistin relative to the treatment with BC1 alone (Fig. 3). A similar trend was also observed for CCCP, which implies that for enhancing intracellular colistin accumulation, both BC1 and salicylate are required and not one compound alone. This indicates the effectiveness of simultaneous inhibition in both MarR and AcrAB-TolC efflux pump.

### Time kill studies

**Figure 4:**
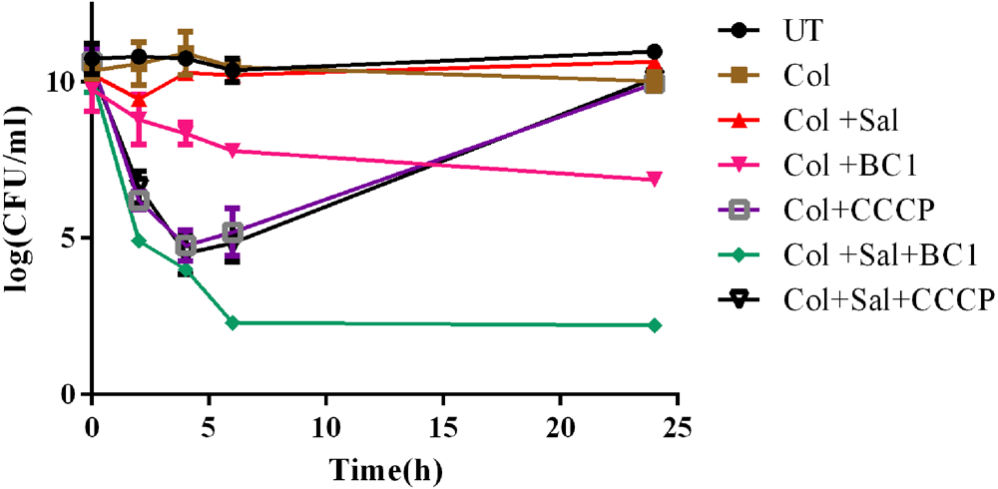
Bactericidal effect of colistin is potentiated by combinatorial use of salicylate (Sal) and BC1. Time kill curve analysis was performed by treatment of early log-phase cells of U3790 with different treatment combinations and the samples from each group were drawn at specific time points from 0–24 h. The samples were serially diluted and plated on to LA plates and incubated at 37 °C. The colony count was expressed as log (CFU/ml). The experiment was performed in triplicates and the error bar represents their standard error of the mean.

Bactericidal effect of colistin along with MarR inhibitor (salicylate) and efflux pump inhibitor (BC1) was evaluated by time kill assay against U3790. Early-log phase culture of U3790 was subjected to different treatments viz., (i) untreated (ii) colistin (iii) colistin + salicylate (iv) colistin + BC1 (v) colistin + CCCP (vi) colistin + salicylate + BC1 (vii) colistin + salicylate + CCCP. The samples were drawn at different intervals, plated onto LB agar and incubated for 24–48 h at 37 °C. The results (Fig. 4) revealed that for U3790 strain, both colistin, and colistin + salicylate treatment were ineffective and no significant reduction in cell counts was observed between untreated, colistin treated and colistin + salicylate treated groups by 24 h. Whereas, colistin + BC1 treatment caused a significant ~3 log fold reduction in cell counts relative to the initial population, implying synergy of BC1 with colistin, mirroring the results of the checkerboard assay. When colistin was combined with both salicylate and BC1, a drastic reduction in cell counts of ~6 log fold was observed and most importantly, no re-growth was observed (Fig. 4), underscoring the potential of MarR inhibitor (salicylate) along with efflux pump inhibitor (BC1) in mitigating the growth of colistin resistant U3790 strain. Colistin + CCCP and colistin + salicylate + CCCP showed a reduction of ~4 and ~6 log fold, respectively, at 6 h but at 24 h, re-growth was observed in CCCP treatment combinations, indicating Col + Sal + BC1 was more effective in restoring colistin sensitivity than Col + Sal + CCCP.

### Fish toxicity studies

**Figure 5:**
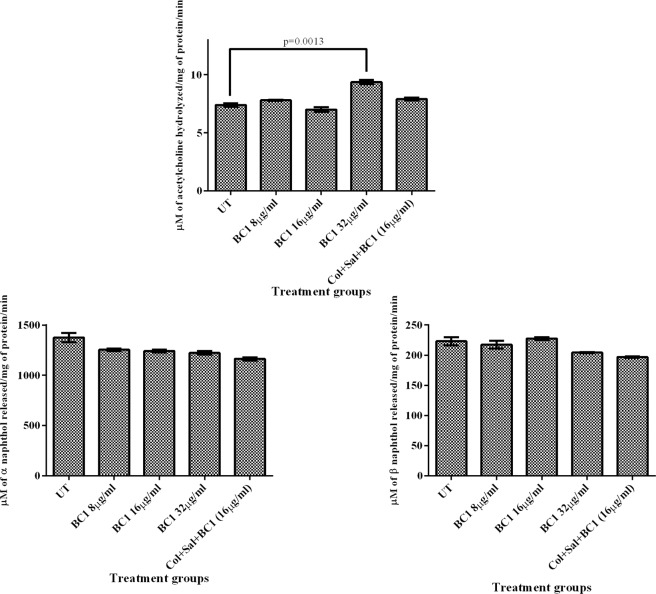
BC1 and Triad combination (colistin + salicylate + BC1) were non – toxic to zebrafish. Zebrafish were injected intramuscularly with (a) 0.5X (b) 1X (c) 2X MEC of BC1 (d) Colistin + Sal + BC1. Liver and brain tissues were isolated by dissection from euthanized fish after 48 h and tested for liver (alpha and beta naphthol) and brain (acetylcholine esterase) enzyme activities. The experiment was performed in triplicates and statistical analyses were performed using Student’s t-test.

Since colistin and salicylate are already in therapeutic use, only BC1and combination of colistin + salicylate + BC1 (triad) were evaluated for its toxicity in zebrafish by quantifying the brain and liver enzyme profiles due to BC1/triad treatment. CCCP (10 μM) was also evaluated for its toxicity in zebra fish. The results (Fig. 5) showed that the treatment of fish with 2X Minimum effective concentration (MEC) of BC1 (32 µg/ml) was non-toxic to the liver as the levels of liver alpha and beta naphthol were comparable to that of the untreated control. As regards brain enzyme profiles at 2X MEC, BC1 induced a discernible elevation in acetylcholine esterase levels relative to untreated control which was statistically significant with P = 0.0013. But at 0.5X and 1X MEC of BC1, no difference in brain acetylcholine esterase levels relative to untreated control were observed, indicating that at the optimized 1X MEC dose of 16 µg/ml, BC1 is non- toxic to zebrafish. Fish injected with CCCP died before 24 h, proving its toxic nature. When triad combination was injected in zebra fish and evaluated for its toxicity, no significant differences in brain and liver enzyme profiles were observed between untreated and triad treated groups, implying that the combination is non-toxic in zebra fish.

### *In vivo* infection study

**Figure 6:**
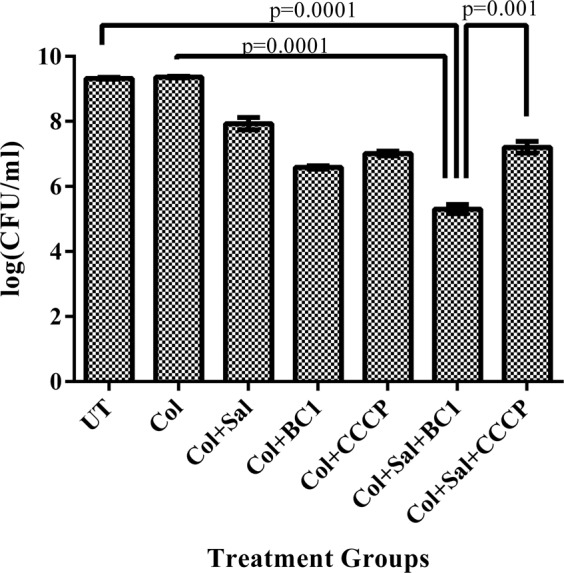
Triad combination (colistin + salicylate + BC1) effectively reduces bacterial bioburden in infected zebrafish. 10^5^ CFU/ml of U3790 was injected intramuscularly in zebrafish. Different treatment combinations were administered 2–3 h post-infection. The zebrafish were sacrificed after 48 h and the colony counts (CFU/ml) from dissected muscle tissue was determined by plating the samples on agar plates. Experiment was performed in triplicates and statistical analyses were performed using Student’s t-test.

Zebrafish were injected intramuscularly with 12 h grown culture of U3790 that was diluted to 0.2 OD (~1 × 10^6^ CFU/ml). 2 h post infection, fish was subjected to various treatments via intramuscular injection: (i) colistin (ii) colistin + salicylate (iii) colistin + BC1 (iv) colistin + salicylate + BC1. 24 h post treatment, fish was euthanized, the muscle tissue was dissected, homogenized, serially diluted and plated on to LB agar plates. 24–48 h post incubation, colony counts were determined (Fig. 6). The untreated (infected) group exhibited ~9 log CFU/ml for both strains. No decline in cell counts was noted for colistin treated group as colistin was used at 1/8 MIC (4 µg/ml) (Fig. 6). Treatment with colistin (4 µg/ml) + salicylate (5 mM) resulted in 1.4 log CFU decline in U3790. The administration of colistin (4 µg/ml) + BC1 (16 µg/ml) resulted in a further 2 log fold decline relative to colistin + salicylate treatment. Finally, treatment with colistin + salicylate + BC1 resulted in a 4 log decline in bioburden relative to untreated/colistin treated fish. Colistin + CCCP and colistin + salicylate + CCCP resulted in a 2 log reduction. The reduction in bioburden due to triad treatment (colistin + salicylate + BC1) was highly significant (P < 0.01) relative to either untreated control/ colistin treatment/ Col + Sal + CCCP treatment as determined by the student’s t-test (Fig. 6). Thus, a combination of MarR inhibitor (salicylate) in conjunction with efflux pump inhibitor (BC1) was highly effective *in vivo* than Col + Sal + CCCP in colistin resistant strain of *E. coli*, which affords credibility for screening this combination in mice sepsis model.

## Discussion

Carbapenem resistant *Enterobacteriaceae* (CREB) has become a major concern for public health due to the production of β-lactamases, carbapenemases, expression of multidrug efflux pumps and alteration in penicillin binding proteins (PBP)^3,25–28^. WHO has also declared CREB as a critical priority pathogen needing urgent attention in the form of new antimicrobials or resistance modulatory agents to tackle CREB infections. A two year study was carried out to assess the prevalence of CREB in rural regions of southern India showed a higher prevalence of carbapenem resistant strains of *E. coli* and *Klebsiella* spp, which emphasized the urgent need to develop necessary control measures and timely detection of CREB infections in the region^29^. Based on recent DeNIS study carried out in 3 neonatal tertiary care centers in New Delhi, it was observed that apart from *Acinetobacter baumannii*, both *K. pneumoniae* and *E. coli* are the major cause of neonatal sepsis and importantly 54% of *K. pneumoniae* strains and 38% of *E. coli* strains are multidrug resistant resulting in high mortality^30^.

CREB infections are typically treated with colistin, which is regarded as a last resort drug^7,31^. Widespread dissemination of colistin resistant phenotype through *mcr* 1–9 plasmid in *Enterobacteriaceae*^8,32–34^ has severely hampered treatment options for CREB. Hence, there is an urgent need to search for alternative therapy to restore colistin sensitivity in colistin resistant *Enterobacteriaceae*. In our previous study^17^, we have shown that pectin capped platinum nanoparticles cures plasmid of the same strain (U3790) and restores sensitivity to meropenem *in vitro* and *in vivo*. In the present study, we used a combination of a MarR inhibitor and an appropriate efflux pump inhibitor to restore colistin sensitivity in the same strain.

Whole-genome sequencing and further MLST analysis using the CGE server revealed that the strain belongs to sequence type ST405 and is closely related to another clinical isolate UMN026 (NC_011751.1) with template coverage of 0.65. ST405 was previously reported as a multi drug resistant uropathogenic *E. coli*, isolated from urine of a patient, which was known to possess composite IS26 transposon that harbored multiple antibiotic resistant genes flanked by mobile IS26 transposons in a unique chromosomal location *yjdA*^35^. Another ST405 MDR *E. coli* was also shown to exhibit persistent carriage and prolonged infection in a patient admitted to the bone marrow transplant unit of tertiary care hospital in Italy^36^. A recent study from China showed that MDR *E. coli* ST405 that belonged to phylogroup D, was cryptically transmitted within a hospital and sequencing revealed that strain harbored uncommon NDM-4 that was flanked by IS26 element, implying potential to mobilize NDM-4 gene^18^. In a recent report, increased expression of *acrA* and *mdfA* was observed to correlate with levofloxacin resistance in 28 *E. coli* clinical isolates obtained from urine^37^. Similarly, an earlier study has shown that increased expression of *acrA* was observed in all imipenem resistant MDR. *E. aerogens* strains which also conferred resistance to quinolones, tetracycline, and chloramphenicol^38^.

A recent study by Lazar *et al*.^15^ has elegantly shown that drug resistant clinical isolates of *E. coli* harbor mutation in *marR* loci, which de-represses *marA* resulting in antibiotic resistant phenotype, due to overexpression of AcrAB-TolC efflux pump. Furthermore, *marR* de-repression also triggers *waaY* expression through *marA*, which leads to increased cell surface negative charge ultimately resulting in collateral sensitivity to cationic antimicrobial peptides. Interestingly, genome analysis of U3790 revealed that it also harbored *marR* mutation (Supplementary Table 2) but it was different from prominent V84E reported by Lazar *et al*.^15^. Hence, the implications of *marR* mutation in U3790 remain to be explored in further studies. Because colistin also interacts with cell surface by electrostatic attraction, we asked whether small molecule inhibition of MarR would restore colistin sensitivity in colistin resistant *E. coli*. Towards this end, we first measured cell surface charge using zeta potential analyzer in the presence and absence of salicylate, a well-known MarR inhibitor^39^. Our observations (Table 1) showed that MarR inhibition by salicylate induced significant alteration in cell surface negative charge of only colistin resistant strain of *E. coli*, but not in colistin sensitive strains. Moreover, among salicylate untreated strains, colistin resistant strain displayed only half of cell surface negative charge as that exhibited by colistin sensitive strains, which might account for altered susceptibility of resistant strain.

MIC reversal studies also showed that only colistin resistant strains responded well to salicylate mediated MarR inhibition, by exhibiting an 8 fold decline in colistin MIC, whereas colistin sensitive strain displayed only a 2 fold reduction in colistin MIC (Table 2). Thus, our MIC reversal results concurred with zeta potential measurements and showed that salicylate altered cell surface charge and colistin sensitivity predominantly in colistin resistant strains. Although 8 fold MIC reversal in resistant strain due to salicylate treatment was significant, it was not drastic as expected, which could be attributed to the fact that inhibition of MarR by salicylate would trigger *marA* mediated expression of AcrAB-TolC efflux transporter. In the present study, we also observed that BC1, at sub-lethal concentration inhibited EtBr efflux better than positive controls CCCP and PAßN (Fig. 1) and also reduced colistin MIC drastically from 4 to 64 fold in various strains of *E. coli*. Importantly, all colistin resistant strains of *E. coli* used in the present study were re-sensitized to colistin by BC1 way below the CLSI break point of >2 µg/ml. Thus, BC1 alone (at sub lethal levels) by virtue of its ability to inhibit colistin efflux was sufficient to reverse colistin MIC in most colistin resistant strains of *E. coli*.

In U3790 strain, the colistin + salicylate + BC1 combination further reduced colistin MIC by 8 fold to 0.25 µg/ml with an overall 128 fold reduction in colistin MIC from 32 µg/ml to 0.25 µg/ml (Table 2). Thus, the combination of colistin + salicylate + BC1 fully restored colistin sensitivity in colistin-resistant strain and it also caused a significant reduction of colistin MIC in colistin sensitive strains. Hence, this combinatorial approach can help in reducing the dose of colistin in therapy thereby mitigating the adverse effect of colistin. We showed in the recent past that dansylated colistin is an efflux substrate and can be accumulated within cells due to treatment with efflux inhibitors^40^. The triad combination was also able to enhance the intracellular accumulation of dansylated colistin effectively in resistant *E. coli* (Fig. 3). Time kill assay revealed that among various combinations evaluated colistin + salicylate + BC1 was highly effective in reducing cell counts by 6 log fold in U3790 strain with no evidence of re-growth even by 24 h.

*In vivo* testing of combinations in restricting the growth of colistin resistant *E. coli* in infected muscle tissue of zebrafish displayed a trend that resembled *in vitro* time kill assay (Fig. 4). The triad combination (colistin + salicylate + BC1) was highly effective in restricting the growth of U3790 strain with a significant ~4 log reduction in cell counts relative to untreated/ colistin treated cells (Fig. 6). Despite its efficacy, the drastic 6 log reduction in cell counts observed *in vitro* was not noted *in vivo*, which could be due to poor solubility of BC1 in the aqueous intracellular milieu of pH 7.2. Future efforts would involve making derivatives of BC1 that display better solubility in aqueous environments while retaining its potent efflux inhibitory potential. Although CCCP was equally effective *in vitro* in time kill assay, it displayed re-growth by 24 h (Fig. 4) and in zebrafish infection study it displayed only a 2 log reduction (Fig. 6). Above all, CCCP being a protonophore is known for its toxicity^41^. Moreover, fish administered with CCCP at recommended concentration died within 24 h proving the toxicity of CCCP. Thus, BC1 was superior to CCCP both in terms of reduced toxicity and enhanced *in vitro* and *in vivo* efficiency in potentiating colistins’ bactericidal effect (Fig. 5).

Synergy of colistin with other antimicrobials have been widely reported earlier against different bacteria^42,43^. In a murine thigh infection model of infection with 12 XDR *A. baumannii* isolates, it was shown that relative to colistin monotherapy, colistin-rifampicin, and colistin –fusidic acid were effective in curtailing bacterial bioburde*n in vivo*. Colistin-meropenem was effective in strains where MIC of meropenem was <32 µg/ml^44^. To mitigate plasmid-borne (*mcr*-1 mediated) colistin resistance, it was shown that colistin permeabilizes the outer membrane of gram-negative bacteria and potentiates the effect of gram-positive specific antimicrobials on gram-negative bacteria. By this strategy, colistin-clarithromycin combination was quite effective against colistin resistant *K. pneumoniae* in murine thigh infection model and bacteremia infection model^45^. Because colistin has adverse side effects, to initiate its reuse in a full-fledged manner, it is important to reduce colistin dosage to mitigate its toxicity. In the present study, by using a combination of MarR inhibitor (salicylate) along with efflux pump inhibitor (BC1), colistin was employed at 1/8 of its MIC, for zebrafish infection study wherein colistins’ bactericidal effect is potentiated and its adverse effects can be abrogated successfully.

## Materials and Methods

### Strains and compounds

*Escherichia coli* K-12 MG1655 was obtained as a.pngt from Dr. Aswin, NCBS, Bangalore. The clinical isolates of *E. coli* – U3790, U3176, U1024, U2354, and U1007 were obtained from Sundaram Medical Foundation (SMF), Chennai, India. Enteropathogenic strain IDH 09519 and enterotoxigenic strain IDH 07933 were kindly.pngted to us by Dr. T. Ramamurthy, THSTI, Faridabad, Haryana. The strains were sub-cultured from glycerol stocks and plated on TSA. Antibiotics and chemicals used in the study were purchased from Sisco Research Laboratories Pvt. Ltd., (SRL), India or HiMedia, India. Benzochromene derivative (BC1) was synthesized using polyethyleneimine (PEI) catalysis as reported earlier^14^. The stocks of the compounds and antibiotics were freshly prepared in respective solvents and stored in −20 °C.

### Antibacterial studies

The minimum inhibitory concentration (MIC) of colistin, BC1 and salicylate were determined for the reference strains and clinical isolates of *E. coli* using micro broth two-fold dilution method^46^. The antibiotic and the compounds were serially diluted from 128 μg/ml to 1 μg/ml using Muller-Hinton broth (Cation adjusted) and inoculated with respective cultures. MIC was determined by measuring OD at 595 nm, after incubation for 18–24 h at 37 °C.

### Whole genome sequencing

WGS was performed using Illumina HiSeq 2500 in high throughput run mode using 2 × 125 bp format. The sequencing library was prepared using TrueSeq DNA library sample prep kit v2 following the manufacturer’s guidelines. The raw read sequence was submitted to the SRA database of NCBI (National Center for Biotechnology Information) with accession number PRJNA541219. The raw reads were assembled by BWA program using *Escherichia coli* K-12, as the reference genome. The assembled genome was annotated using RAST (Rapid Annotations using Subsystems Technology) database. To identify the resistance contributing genes, the complete sequence was submitted in CARD and ResFinder (Centre for Genome Epidemiology). Sequence type was identified using MLST database of the Center for Genomic Epidemiology. To discern the effect of expression efflux pump genes in colistin resistant U3790, we performed semi-quantitative gene expression studies by amplifying *acrA, acrB* and *tolC* genes in U3790. We used colistin sensitive U3176 and reference strain MG1655 for comparison. Total RNA from the bacterial strains were isolated using Aurum™ total RNA mini kit (Bio Rad). cDNA conversion was done using the iScript™ cDNA synthesis kit (Bio Rad). Both RNA and cDNA was quantified using Qubit fluorimeter (Invitrogen). Equal concentrations of cDNA was used as template for the reaction. The obtained PCR product was quantified using ImageJ software. The intensity of the band from clinical strains were normalized with those of reference strain and represented as relative fold change in expression.

### Zeta potential measurement

The alteration in surface charge of bacteria on treatment with salicylate was studied by measuring the zeta potential as reported earlier^22^. 1 × 10^9^ cells were harvested by centrifugation and resuspended in Milli-Q™ water. Salicylate (5 mM) was provided to the culture and incubated for 20 min, after which the cells were harvested, resuspended and diluted 10 fold. Respective untreated controls were maintained. The electrophoretic mobility was measured using Zetasizer Nano ZS™; Malvern Instruments Ltd, UK. The electrodes were washed extensively using ethanol and water after measurement.

### EtBr accumulation and efflux studies

Early log phase cells of *E. coli* were harvested in buffer with 0.2% glucose and allowed to incubate with 10 µg/ml ethidium bromide at 37 °C for 30 min. BC1, BC6, and BC9 were added at 16 µg/ml^47^. Fluorescent intensity was measured for 25 min in an interval of 5 min at Ex 530 nm and Em 585 nm. For efflux studies, early log phase cells of resistant *E. coli* were de-energized with CCCP, washed after 20 min and harvested^48^. The cells were then treated with different Benzochromene derivatives and ethidium bromide. CCCP and PAßN were used as positive controls. After incubation for an hour, the cells were washed and harvested in buffer containing glucose to re-energize them. Efflux was measured by recording fluorescence at for 25 min at 5 min intervals. The experiments were done in triplicates and represented as average value with standard error of the mean.

### Membrane permeability assay

To evaluate if BC1 has membrane permeabilizing property, we performed NPN uptake assay^49^. NPN (1-N-phenylnaphthylamine) is a non-polar fluorophore that exhibits fluorescence in the phospholipid environment. An intact outer membrane of Gram negative bacteria is asymmetric and confers permeability barrier to hydrophobic moieties like NPN. When the outer membrane is permeabilized, NPN gains access and exhibits fluorescence. Mid log cells of U3790 were treated with BC1. Colistin and CTAB were used as controls. The fluorescence of NPN at Ex 375 nm and Em 420 nm was recorded within 3 min. NPN uptake factor was calculated as the ratio of fluorescent value of background-subtracted bacterial suspension to that of the buffer.

Qualitative analysis of the effect of BC1 on outer membrane integrity was performed by visualizing propidium iodide (PI) uptake using fluorescent microscopy. Mid log cells were treated with either BC1, colistin or CTAB in the presence of PI. The cells were then viewed and imaged using a fluorescent microscope (Nikon Eclipse Ni-U, Japan). The result shows the average value of the triplicates and error bar represents the standard error of the mean.

### Membrane potential studies

To discern whether BC1 inhibited efflux by disrupting membrane potential like CCCP, membrane potential studies were performed as reported earlier^50^. A cationic membrane permeabilizing dye, Disc3, was used for the assay. Disc3 was added to mid log cells of U3790 and fluorescence intensity (Ex 622 nm and Em 670 nm) was recorded until plateau. Subsequently, 0.5% glucose was added and fluorescence was further recorded. BC1 and 10 μM CCCP was added and the change in fluorescent intensity was documented. The experiment was performed in triplicates.

### Checkerboard analysis

The interaction of colistin with salicylate + BC1 was evaluated by checkerboard analysis performed on a 96 well plate, as reported earlier^51^. The combinatorial effect of colistin with salicylate and colistin with BC1 was tested against the reference and the clinical isolates of *E. coli*. CCCP was used as a positive control. The antibiotic was serially diluted along the y-axis from 32–0.25 μg/ml and the compound along the x-axis from 64–0.5 μg/ml. The Fractional Inhibitory Concentration (FIC) was calculated as the Eq. (1):

1 $${\rm{FIC}}\,{\rm{index}}=\frac{{\rm{MIC}}\,{\rm{of}}\,{\rm{drug}}\,{\rm{A}}\,{\rm{in}}\,{\rm{combination}}}{{\rm{MIC}}\,{\rm{of}}\,{\rm{drug}}\,{\rm{A}}\,{\rm{alone}}}+\frac{{\rm{MIC}}\,{\rm{of}}\,{\rm{drug}}\,{\rm{B}}\,{\rm{in}}\,{\rm{combination}}}{{\rm{MIC}}\,{\rm{of}}\,{\rm{drug}}\,{\rm{B}}\,{\rm{alone}}}$$

FIC index ≤0.5, indicates a synergistic effect, between 0.5–2.0, indicated additive effect and ≥2.0 indicates antagonism^52^. The minimum effective concentration (MEC), at which synergy was observed was also determined.

### Resistance modulation assay

The ability of the compounds – salicylate, and BC1 to reverse the antibiotic resistance was discerned by resistance modulation assay^53^ performed in a 96 well plate. The reduction in MIC of colistin against the different clinical isolates, when combined with (i) salicylate (ii) BC1 (iii) salicylate + BC1, (iv) salicylate + CCCP, were determined. This was expressed in terms of the modulation factor, which can be calculated as given by the Eq. (2):

2 $${\rm{Modulation}}\,{\rm{Factor}}=\frac{{\rm{MIC}}\,{\rm{of}}\,{\rm{antibiotic}}\,{\rm{alone}}}{{\rm{MIC}}\,{\rm{of}}\,{\rm{antibiotic}}\,{\rm{when}}\,{\rm{combined}}\,{\rm{with}}\,{\rm{compound}}}$$

The cut off for biologically significant modulation was set as a modulation factor of >2^54^. The concentration of the compounds was determined based on the results of the checkerboard analysis.

### Colistin accumulation studies

To observe the accumulation of colistin with the cells in the presence of salicylate with and without EPI, colistin was dansylated using dansyl chloride as reported earlier^22^. Dansyl group contributes to fluorescence and when conjugated with colistin, enables us to visualize under a fluorescent microscope. Early log phase cells were incubated with colistin-dansyl chloride in the presence and absence of salicylate with and without BC1 for 3 h. CCCP was used as a positive control. The samples were viewed and imaged using a fluorescent microscope (Nikon Eclipse Ni-U, Japan).

### Time kill studies

The reduction of bioburden *in vitro* was assessed by time kill curve analysis as reported earlier^55^. The planktonic cells were grown until the early log phase (0.3–0.4 OD) and were divided into groups that were subjected to various treatments. The samples were drawn at specific time intervals from 0 to 24 h and plated on Luria Bertani agar plates. The viability of cells was evaluated by determining the plate counts after incubation at 37 °C for 24 h and expressed as colony forming units/ml (CFU/ml). Bactericidal activity was discerned, when there is ≥3 log decrease in CFU/ml relative to the initial culture inoculum. The combination can be recognized as synergistic if it shows ≥2 log decrease in CFU/ml, relative to treatment with individual components^56^. All experiments were done in triplicates.

### *In vivo* infection studies

The *in vivo* experiment was performed by strictly adhering to CPCSEA guidelines for laboratory animal facilities (Central Act 26 of 1982). The protocols followed were approved by the Institutional Animal Ethics Committee (CPCSEA-510/SASTRA/IAEC/RPP) of SASTRA deemed University, India. *Danio rerio* (zebra fish) was used as an *in vivo* model for infection studies and was performed as reported earlier^57^. The zebrafish were injected intramuscularly by a 3/10-cc U-100 insulin syringe with a 29-gauge needle. 10 μl of the resistant strain (10^5^ CFU/ml) was injected and was left for 2–3 h for the infection to accomplish. The compounds (Salicylate/Benzochromene/ CCCP) and antibiotic, alone and in combination, were then injected and the fish were monitored for 24–48 h. After 48 h, the fish were euthanized, sacrificed by decapitation, dissected and the muscle tissue was isolated. The tissue was homogenized, serially diluted and plated on to Luria Bertani Agar plates. The colony counts were evaluated after incubation at 37 °C for 24 h and the reduction in bioburden was analyzed. Experiments were performed in triplicates.

### Statistical analyses

All experiments in the study were performed in triplicates as individual experiments. Statistical analyses were performed by Student’s t-test using Graph Pad Prism version 7.0 for Windows (Graph Pad Software Inc., San Deigo, CA, USA).

#### Conclusion

In summary, we performed whole-genome sequencing of colistin resistant strain that revealed efflux is a major contributor to drug resistance in this strain and colistin resistance is probably mediated by the L-Ara-4-N pathway. Furthermore, we were successful in re sensitizing colistin resistant *E. coli* by a synergistic combination of colistin with MarR inhibitor (Salicylate) that enhances colistin binding and efflux pump inhibitor (BC1) that prevents colistin extrusion. This triad combination was highly effective in reducing cell counts by a drastic 6 log fold in U3790 by time kill assay. The *in vivo* studies added proof to the triad’s bactericidal effect by causing a remarkable ~4 log fold decline in cell counts for U3790, highlighting the efficacy of this combinatorial approach. Based on promising results obtained, this combination deserves to be tested in the mice sepsis model. To achieve re sensitizing effect observed *in vitro*, future studies would also aim to make improved derivatives of BC1, that display enhanced solubility in aqueous milieu while retaining its remarkable efflux inhibitory potential.

## Supplementary information

Supplementary Information

## Data Availability

Almost all data generated or analyzed during this study are included in this published article (and its Supplementary Information files). The raw data would be available upon request.
