# Supplementary material for: Restoring colistin sensitivity in colistin-resistant E. coli: Combinatorial use of MarR inhibitor with efflux pump inhibitor
Source: Sci Rep. 2019 Dec 25;9:19845. doi: 10.1038/s41598-019-56325-x (PMC6934491; doi:10.1038/s41598-019-56325-x)

**Supplementary Information:**

**Restoring colistin sensitivity in colistin resistant *E. coli*: Combinatorial use of marR inhibitor with efflux pump inhibitor**

Niranjana Sri Sundaramoorthy^a^, Pavithira Suresh^b^, Subramaniapillai Selva Ganesan^b^, ArunKumar GaneshPrasad and Saisubramanian Nagarajan^a^*

^a^ Center for Research on Infectious Diseases, School of Chemical and Biotechnology, SASTRA Deemed to be University, Thanjavur, Tamil Nadu, India.

^b^ Department of Chemistry, School of Chemical and Biotechnology, SASTRA Deemed to be University, Thanjavur, Tamil Nadu, India.

***Corresponding Author**

**Dr. SaisubramanianNagarajan**

Center for Research on Infectious Diseases

ASK-I-312, SCBT, SASTRA

Thanjavur- 613401

Email: sai@scbt.sastra.edu

**Supplementary Table 1: Antimicrobial profiling of different strains of *E. coli***

| **Strains** | **Minimum Inhibitory Concentration (μg/ml)** | | | |
| --- | --- | --- | --- | --- |
|  | **Colistin** | **Salicylate** | **BC1** | **CCCP** |
| U3790 | 32 | >512 | >256 | 128 |
| U1007 | 4 | >512 | >256 | 128 |
| U2354 | 2 | >512 | >256 | 128 |
| U1024 | 2 | >512 | >256 | 128 |
| IDH09519 | 2 | >512 | >256 | 128 |
| IDH07933 | 2 | >512 | >256 | 128 |
| MG1655 | 2 | >512 | >256 | 128 |
| U3176 | 1 | >512 | >256 | 128 |

**Supplementary Table 2a: Genes implicated in antimicrobial resistance identified by RGI (Resistance Gene Identifier)** tool of CARD database

| **Resistance mechanisms** | **Corresponding genes/mutations present in U3790** |
| --- | --- |
| **Antibiotic efflux** | *tolC, msbA, mdtH, mdtG, marA, evgA, emrY, emrR, emrK, emrB, baeR, YojI, mdtP, mdtO, mdtF, mdtE, mdtC, mdtB, kdpE, gadX, gadW, evgS, mdfA, emrE, acrA, emrA, CRP, cpxA, baeS, acrS, acrF, acre, acrD, acrB* |
| **Efflux and antibiotic target alteration** | *marR* mutation ( K62R, G103S, Y137H), *acrR* mutation, *soxR* mutation, *soxS* mutation |
| **Efflux and reduced permeability to antibiotic** | *marA* |
| **Antibiotic inactivation** | *ampC* beta lactamase |
| **Antibiotic target alteration** | Mutations in PBP3, EF-Tu, *parC, cyaA, glpT, nfsA, gyrA, ugd, pmrF, eptA, bacA* |

**Supplementary table 2b: RGI identified the following antibiotic class for which colistin resistant clinical isolate of *E. coli* (U3790) displays resistance**

| **RGI criteria** | **Antibiotics** |
| --- | --- |
| **Strict** | Rhodamine, carbapenem, nucleoside, tetracycline, aminocoumarin, triclosan, fosfomycin, acridine dye, peptide, fluoroquinolone, pheincol, rifamycin, nybomycin, aminoglycoside, benzalkonium chloride, elfamycin, cephamycin, monobactam, cephalosporin, nitrofuran, glycylcycline |
| **Perfect** | Phenicol, carbapenem, tetracycline, rifamycin, macrolide, cephamycin, triclosan, aminoglycoside, fosfomycin, fluoroquinolone, monobactam, cephalosporin, glycylcycline, nitroimidazole, aminocoumarin |

**Supplementary Table 3: Mutations in Colistin resistant determinant genes of U3790 based on genome analysis**

| **Gene** | **Mutations in U3790** | **Literature Reported** |
| --- | --- | --- |
| *mgrB* | No mutation |  |
| *pmrA* | S29G, G144S | **Serine (AGC) to glycine (GGC)**  Threonine (ACA) to Serine (AGC) |
| *pmrB* | H2R, D286G | **Histidine(CAT) to CGT (Arginine)**  Glutamic acid (GAA) to Aspartic acid (GAT)  Aspartic acid (GAC) to Glycine (GGC)  Valine (GTA) to ATA (isoleucine) |
| *pmrC* | A137T, D338G |  |
| *pmrH* | K69E, R104H, V251A, Q265L, D298N, H368R |  |
| *pmrG* | I14F |  |
| *phoP* | I44L |  |
| *phoQ* | No mutation |  |
| *ugd* | A125V, M185T |  |
| *arnA* | A21S, D77E, D204E, P249S, S252P, K253A, T311A, H627R, Q639H |  |
| *arnT* | A116T, S197T, D232N, M248L, L261V, A281T, P322S, S408P, N522D |  |
| *arnC* | K32T |  |
| *Crr* | No mutation |  |
| *parC* | S80I |  |

**Supplementary Table 4: Genes regulated by m**arA

| **Physiological function** | **Genes** |
| --- | --- |
| Energy metabolism, carbon | *aceE*, *aceF*, *ackA*, *acnA*, *aldA*, *fumC*, *glpD*, *gltA*, *mdaA*, *ndh*, *pflB*, *pgi*, *zwf* |
| Biosynthesis of cofactors, carriers | *accB*, *cobU*, *hemB*, *gshB*, *ribA*, *ribD* |
| Carbon compound catabolism | *galK*, *galT* |
| Amino acid biosynthesis and metabolism | *tnaA*, *tnaL* |
| Fatty acid biosynthesis | *fabB* |
| Nucleotide biosynthesis | *guaB*, *purA* |
| Adaptation | *inaA* |
| Transport/binding proteins | *gatA*, *gatC*, *fecA*, *mglB*, *mtr*, *srlA2*, *tolC, yadG*, *yadH*, *ydeA*, b3469 |
| Protection responses | *acrA*, *marA*, *marB*, *marR*, *nfnB*, *sodA*, *tpx* |
| Cell envelope | *ompF*, *ompX* |
| Ribosome constituents | *rimK*, *rplE* |
| Macromolecule synthesis, modification | *map* |
| Not classified | b0357, b0447, b0853, *mdaB*, *yhbW* |
| Encoding unknown proteins | b1448, b2530, b2889, b2948, *ybjC*, *yfaE*, *yggJ* |

**Supplementary Table 5: Effect of mutations in Master Regulators (*acrR, soxR and soxS*) on Drug resistance**

| **Master**  **Regulators**  **(MR)** | **AMR Related functions affected by MR** | **Drug class** | **Resistance mechanism** |
| --- | --- | --- | --- |
| ***acrR*** | resistance-nodulation-cell division (RND) antibiotic efflux pump | macrolide antibiotic, fluoroquinolone antibiotic, monobactam, aminoglycoside antibiotic, carbapenem, cephalosporin, glycylcycline, penam, tetracycline antibiotic, acridine dye, aminocoumarin antibiotic, rifamycin antibiotic, diaminopyrimidine antibiotic, phenicol antibiotic, triclosan, antibacterial free fatty acids | antibiotic target alteration, antibiotic efflux |
| ***soxR*** | ATP-binding cassette (ABC) antibiotic efflux pump, major facilitator superfamily (MFS) antibiotic efflux pump, resistance-nodulation-cell division (RND) antibiotic efflux pump | macrolide antibiotic, fluoroquinolone antibiotic, monobactam, aminoglycoside antibiotic, lincosamide antibiotic, carbapenem, fosfomycin, cephalosporin, glycylcycline, bicyclomycin, penam, nucleoside antibiotic, tetracycline antibiotic, peptide antibiotic, acridine dye, oxazolidinone antibiotic, aminocoumarin antibiotic, rifamycin antibiotic, diaminopyrimidine antibiotic, phenicol antibiotic, isoniazid, pleuromutilin antibiotic, triclosan, benzalkonium chloride, rhodamine, antibacterial free fatty acids, nitroimidazole antibiotic | antibiotic target alteration, antibiotic efflux |
| ***soxS*** | ATP-binding cassette (ABC) antibiotic efflux pump, major facilitator superfamily (MFS) antibiotic efflux pump, resistance-nodulation-cell division (RND) antibiotic efflux pump, General Bacterial Porin with reduced permeability to beta-lactams | macrolide antibiotic, fluoroquinolone antibiotic, monobactam, aminoglycoside antibiotic, lincosamide antibiotic, carbapenem, fosfomycin, cephalosporin, glycylcycline, cephamycin, bicyclomycin, penam, nucleoside antibiotic, tetracycline antibiotic, peptide antibiotic, acridine dye, oxazolidinone antibiotic, aminocoumarin antibiotic, rifamycin antibiotic, diaminopyrimidine antibiotic, phenicol antibiotic, isoniazid, pleuromutilin antibiotic, triclosan, penem, benzalkonium chloride, rhodamine, antibacterial free fatty acids, nitroimidazole antibiotic | antibiotic target alteration, antibiotic efflux, reduced permeability to antibiotic |


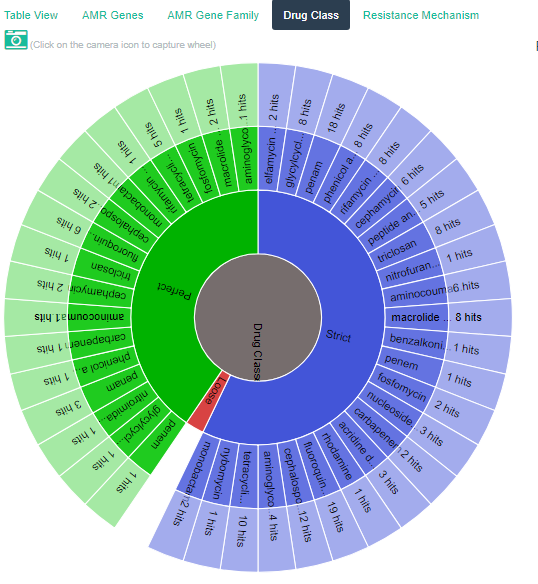
**Supplementary Figure 1: Drug Resistance Profile of U3790 predicted by CARD database based on Genome sequence data** A) RGI wheel format of Strict (> 95% identity) and Perfect Matches (>99% identity) of Antibiotic resistance gene determinants identified from genome of U3790 by Resistance Gene Identifier tool of CARD database

B) The expression of *acrA*, *acrB* and *tolC* was evaluated in colistin resistant U3790, colistin sensitive U3176 and reference strain MG1655. The gene expression is displayed as relative fold change with respect to the expression of the genes in reference strain.

A)

B)

**Supplementary Figure 2: Cart wheel assay shows efflux inhibitory activity of BC1.** Cells of colistin resistant *E. coli* -U3790, U1007, colistin sensitive *E. coli* – U3176 and reference strain MG1655 were swabbed in cart wheel pattern in plates containing 0.1 μg/ml EtBr with or without benzochromene derivatives. CCCP was used as positive control. The plates were incubated at 37ᵒC for 24 h and imaging was performed using UV Transilluminator. Fluorescence were observed in plates containing BC1 and CCCP, proving the efflux pump inhibitory activity of BC1, qualitatively.


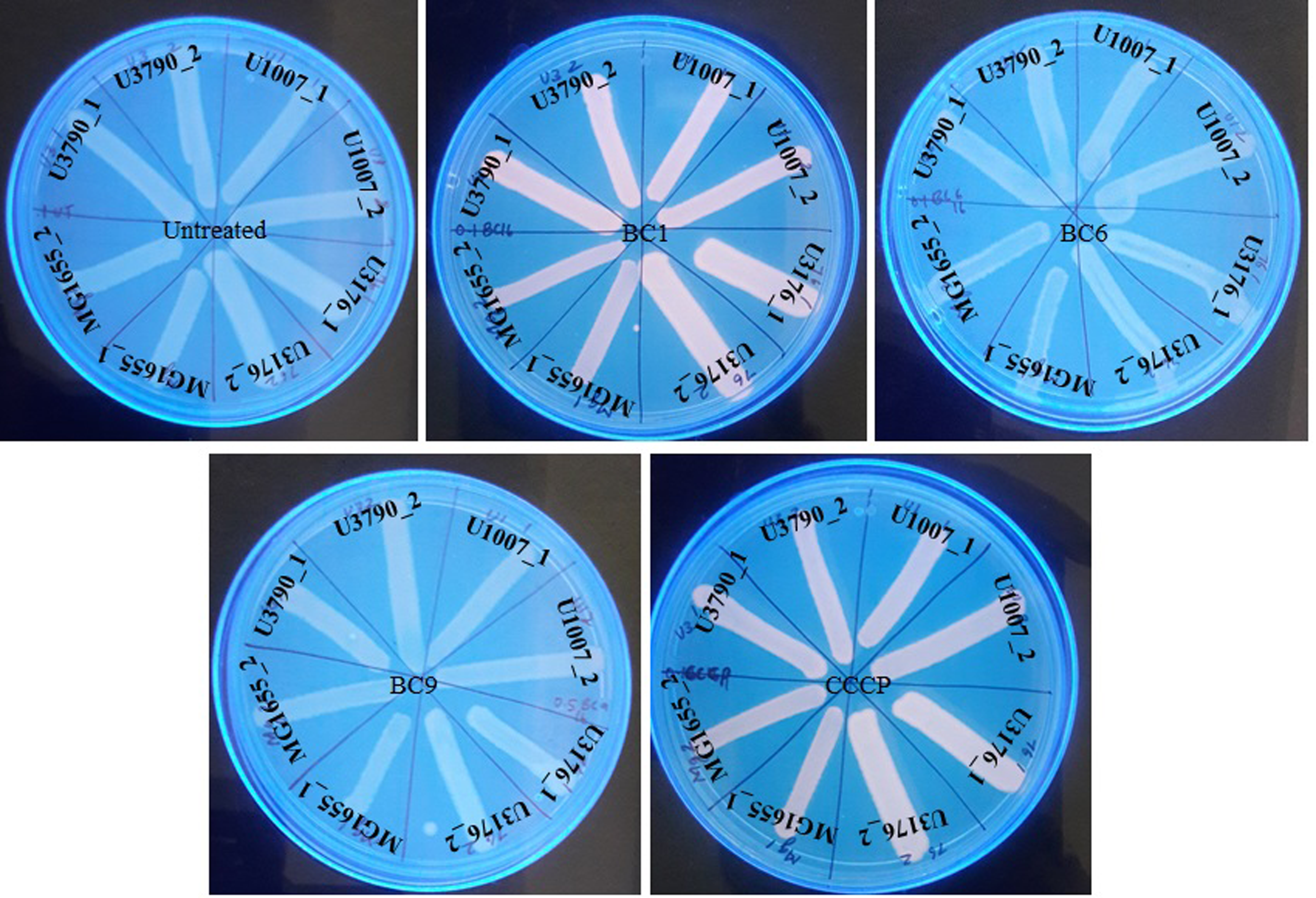


**
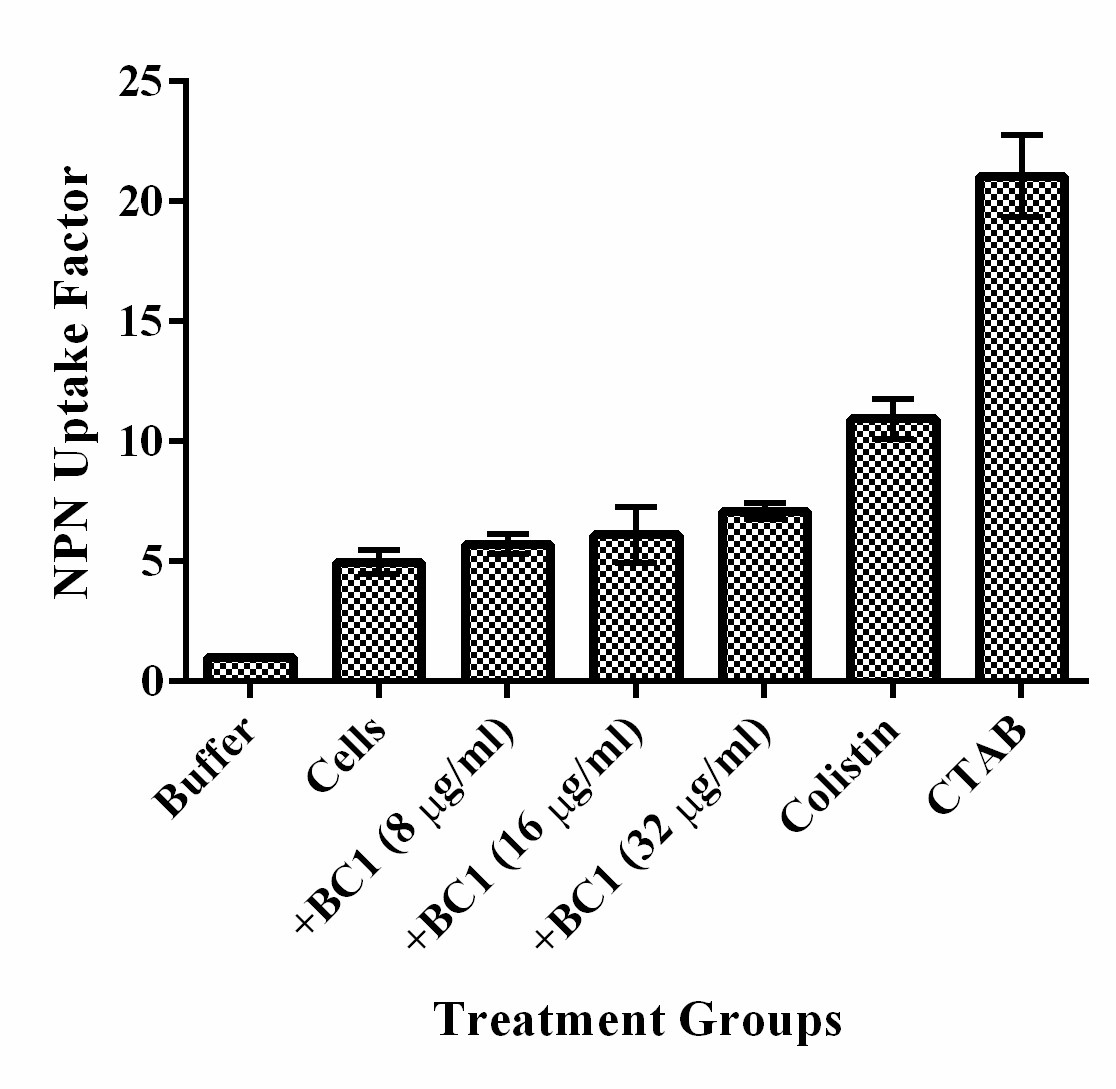
Supplementary figure 3: BC1 does not perturb outer membrane/Inner Membrane permeability in Colistin resistant *E. coli* (U3790).** A) NPN uptake assay: Mid log cells were treated with BC1, colistin and CTAB in the presence of NPN and fluorescence was recorded immediately at Ex375nm and Em420nm. NPN uptake factor was calculated. The error bar represent the standard error of the mean from three independent experiments. B) PI assay: Mid log cells were treated with BC1, colistin and CTAB in the presence of PI and imaged under fluorescent microscope.

A)


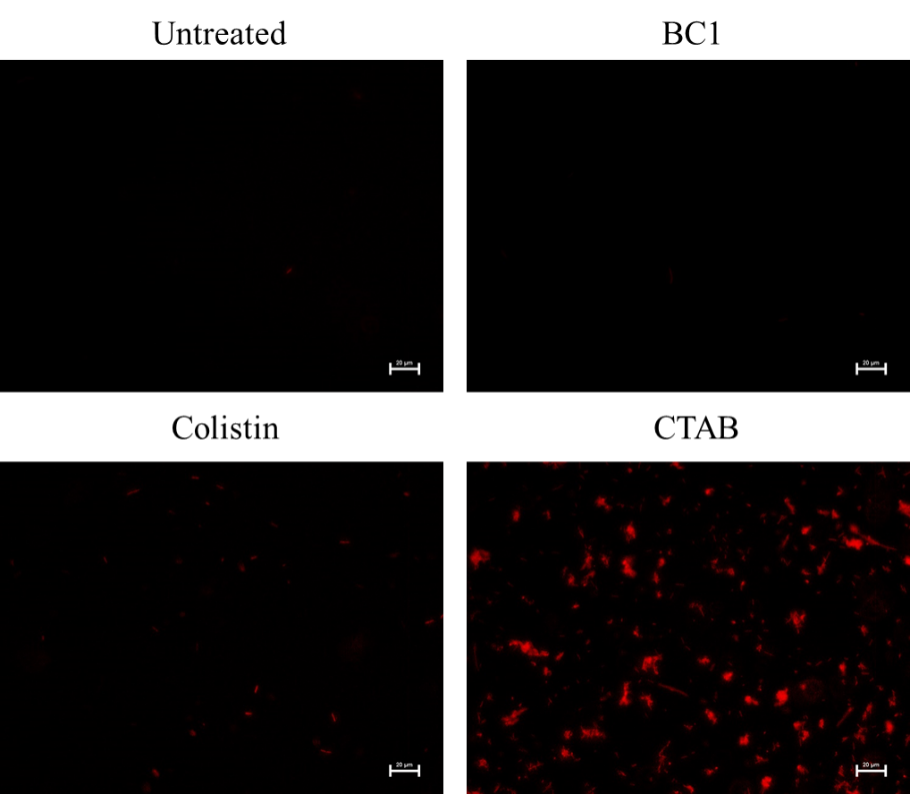


B)

**Supplementary Figure 4: BC1 perturbs the membrane potential of U3790.** Mid log cells of U3790 were harvested, washed and resuspended in 5mM HEPES buffer. Cells were treated with BC1 for 5 min and then Disc3 was added. The fluorescence was measured at Ex622nm and Em670nm. The cells were energized with 0.5% glucose and fluorescence was recorded till the stabilization of DiSc3 fluoresence. The experiment was performed in triplicates and the error bar represents the standard error of the mean.


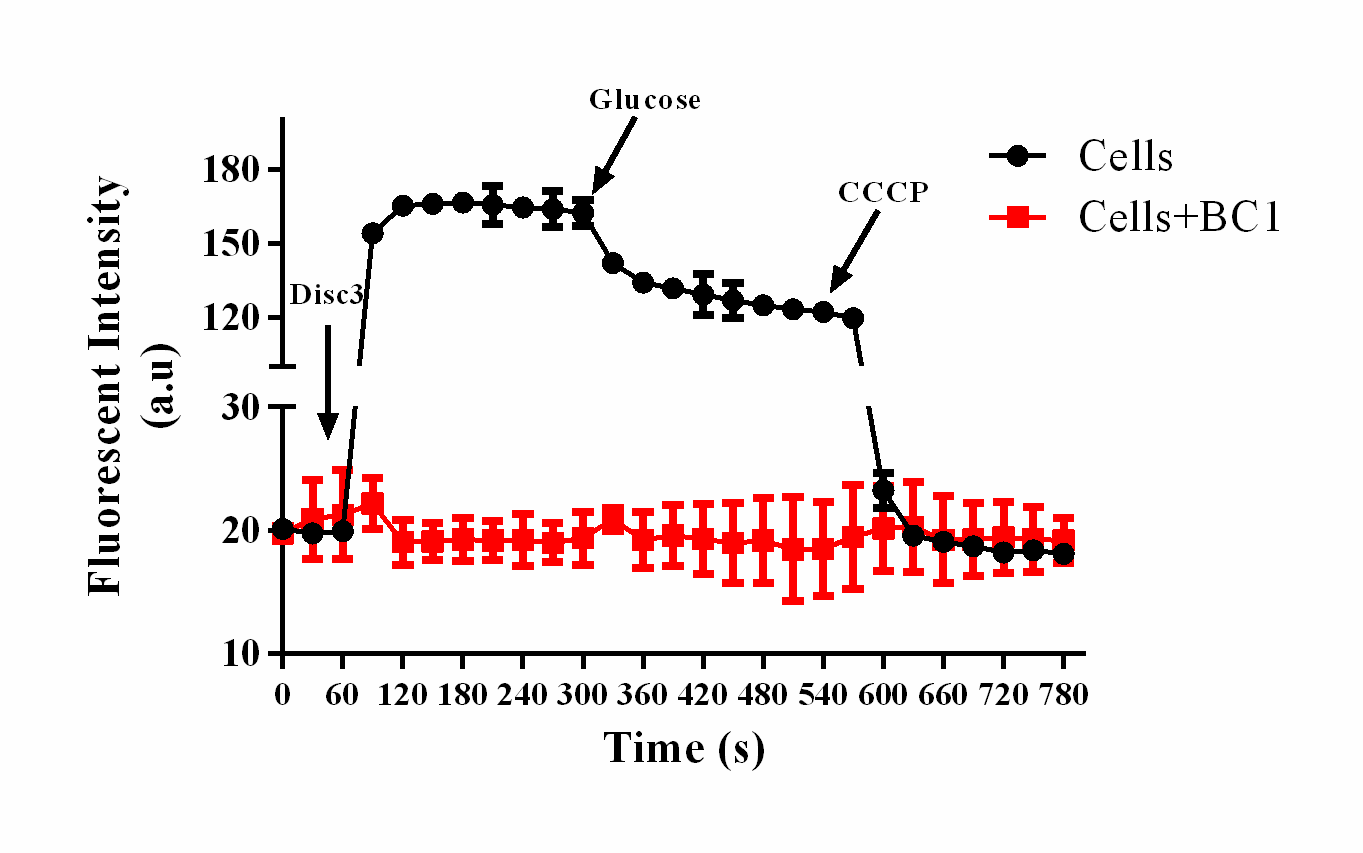

Supplement: Supplementary file 1 — Supplementary Information [file 41598_2019_56325_MOESM1_ESM.doc]
